# BRAF and MEK inhibition beyond dabrafenib–trametinib in advanced thyroid cancer: a real-world case series

**DOI:** 10.3389/fendo.2026.1694805

**Published:** 2026-02-05

**Authors:** Tzahi Yamin, Oded Cohen, Eyal Robenshtok, Elena Izkhakov, Mor Miodovnik, Orit Gutfeld, Yasmin Leshem, Roman Meirovitz, Anton Warshavsky, Nidal Muhanna, Inbar Finkel

**Affiliations:** 1 Department of Otolaryngology, Head and Neck Surgery, Assuta Samson Ashdod Medical Center, Ashdod, Israel; 2 Endocrinology and Metabolism Institute and Davidoff Cancer Center, Rabin Medical Center, Petah Tikva, Israel; 3 Institute of Endocrinology, Metabolism and Hypertension, Sourasky Medical Center, Tel Aviv, Israel; 4 Oncology Division, Sourasky Medical Center, Tel Aviv, Israel; 5 Department of Otolaryngology, Head and Neck Surgery and Maxillofacial Surgery, Sourasky Medical Center, Tel Aviv, Israel

**Keywords:** BRAF V600E mutation, papillary thyroid carcinoma, anaplastic thyroid carcinoma, targeted therapy, real-world evidence

## Abstract

**Objective:**

BRAF V600E mutation is the most common and clinically significant genetic alteration in advanced thyroid cancers. This study provides real-world experience with BRAF and MEK inhibitors other than dabrafenib and trametinib in the treatment of advanced thyroid cancers harboring this mutation.

**Methods:**

A case series of four patients with advanced thyroid cancer (three papillary and one anaplastic) treated with various BRAF and MEK inhibitors. All patients had confirmed BRAF V600E mutation.

**Results:**

Among three patients treated with BRAF/MEK inhibitors for radioiodine refractory metastatic PTC, and one patient with ATC, all (100%) demonstrated a partial response (PR) during therapy, yielding an overall response rate (ORR) of 100%. Stable disease was observed in multiple treatment phases, contributing to a high overall disease control rate. Three patients had disease-related death, while one remained under treatment at last follow-up. The course of treatment was complicated by significant toxicities, leading to dose reductions or treatment discontinuations. Despite initial responses, all cases eventually progressed, necessitating sequential treatment strategies. Overall survival ranged from 6.0 to 25.3 months, with a median follow-up of 18.3 months since the initiation of BRAF and MEK inhibitors.

**Conclusions:**

This case series highlights the potential benefits and challenges of targeted therapies in advanced thyroid cancer. While BRAF and MEK inhibitors offer new treatment options, toxicity management and the development of resistance remain significant hurdles. The limited FDA-approved options for BRAF V600E-positive thyroid cancer compared to melanoma underscore the need for further research to optimize and expand treatment strategies.

## Introduction

Thyroid cancer represents the most common endocrine malignancy, with papillary thyroid cancer (PTC) accounting for approximately 85% of cases (1, 2). While most PTCs have an excellent prognosis, a subset of patients develop aggressive, treatment-refractory disease. At the other end of the spectrum, anaplastic thyroid cancer (ATC) stands as one of the most lethal human malignancies, with a historical median survival of only 3–5 months (3, 4).

The somatic BRAFV600E mutation is present in 37%–60% of PTC, particularly among the tall-cell variants, and to a similar extent in ATC (1–3). The presence of BRAFV600E mutation is linked to dysregulation of the sodium-iodide symporter, reducing the capacity of tumor cells to absorb radioiodine (RAI) and consequently, leading to RAI resistance (4). Patients with BRAFV600E-positive tumors are more likely to exhibit a more aggressive tumor behavior and potentially higher cancer-related mortality, requiring systemic therapies for recurrent or metastatic disease (5, 6).

Recent advances in molecular profiling have revolutionized our understanding of thyroid cancer biology and opened new avenues for targeted therapies (5–7). The identification of driver mutations, particularly the BRAF V600E mutation, has led to the development of targeted tyrosine kinase inhibitors with a promising therapeutic potential in both differentiated and anaplastic thyroid cancers (7–9), extending the overall survival in ATC to 80% at 12 months (8).

To date, the only BRAF/MEK inhibitor regimen approved by the U.S. Food and Drug Administration (FDA) for the treatment of ATC is the combination of dabrafenib and trametinib (10). This approval was initially based on findings from the phase II ROAR basket trial, which demonstrated promising clinical activity, with subsequent research further supporting its efficacy (11, 12). Despite these encouraging results, dabrafenib and trametinib remain the sole labeled combination approved for ATC, in contrast to melanoma treatment, where other BRAF and MEK inhibitor combinations, such as vemurafenib–cobimetinib and encorafenib–binimetinib, are available (13–15). These alternative regimens exhibit slightly different side-effect profiles, which may enable clinicians to modify therapy in the presence of severe adverse events, potentially reducing the need for dose reductions or treatment discontinuation.

**Figure 1:**
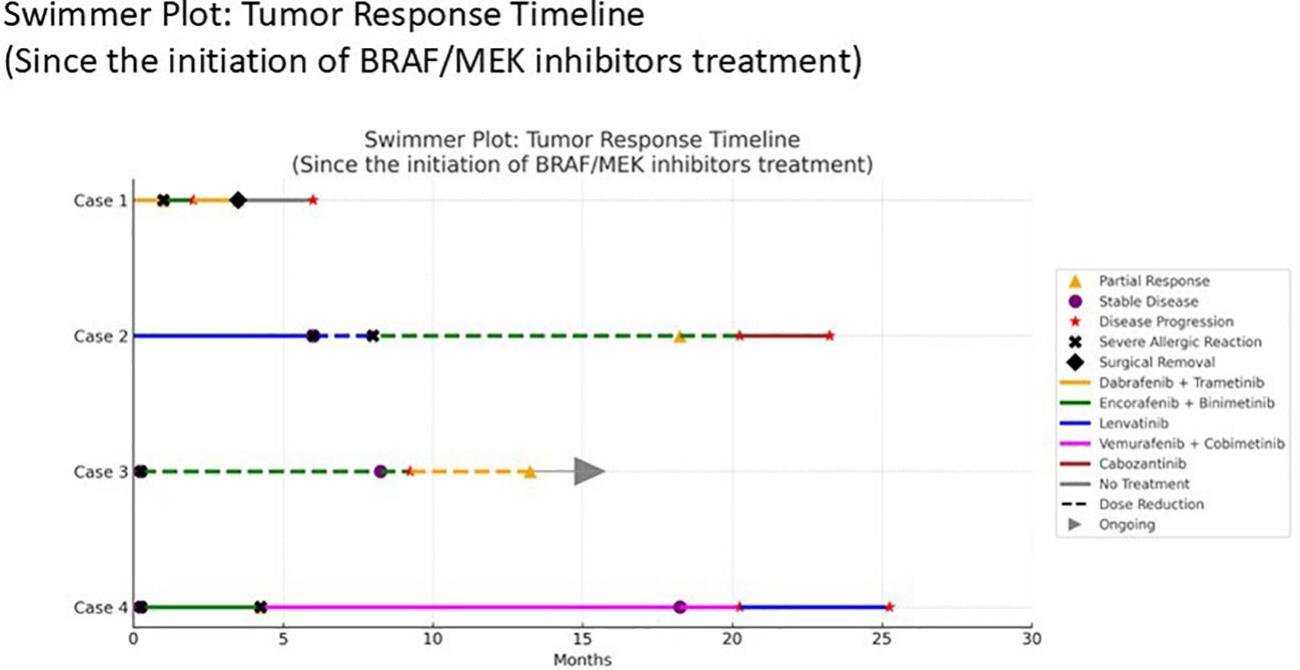
Swimmer plot showing the tumor response timeline since initiation of BRAF/MEK inhibitor treatment. Each row represents an individual patient (cases 1-4). Colored bars denote different systemic therapies administered.

These cases illustrate the complexity of managing advanced thyroid cancer in the era of precision medicine, emphasizing both the benefits and challenges of targeted therapies (13, 16–18). Prior treatments, tumor mutation profiles, and outcomes compared with previously reported cohorts are summarized in **Table 1**, and sequential therapeutic strategies are outlined in **Figure 1**. This series highlights the real-world use of BRAF and MEK inhibitors, management of treatment-related toxicities, and the need for ongoing research to optimize personalized care and improve patient outcomes (14, 23).

**Table 1:** Prior treatments, tumor mutation profiles, and outcomes compared with previously reported cohorts.

Case	N	Age (years)	Variant	Mutation profile	Prior treatments before BRAF + MEK inhibitors	BRAF/MEK inhibitor used	PFS (months)	Dose modification/discontinuation	Grade 3-4 adverse reactions
Case 1	1	68	ATC	BRAF V600E, ATM, TP53	None	D + T; E + B	2.5	Discontinuation	Yes
Case 2	1	67	Tall cell variant PTC	BRAF V600E, TERT, VHL, MSS, TMB 0	Surgery; RAI × 2; radiotherapy; lenvatinib	D + T; E + B	10	Dose reduction; discontinuation	Yes
Case 3	1	83	Tall cell variant PTC	BRAF V600E, MSS, TMB 3.78	Surgery; RAI × 3	D + T; E + B	17	Dose reduction; discontinuation	Yes
Case 4	1	76	Tall cell variant PTC	BRAF V600E, TERT, MEN1 LOF, TMB 1.6	Surgery; RAI × 1	D + T; E + B; V + C	18	Dose reduction; discontinuation	Yes
Brose MS et al. (7)	51	66 (55-74)	PTC	BRAF V600E	VEGFR inhibitor; chemotherapy	V	18.4	Dose reduction (53%); discontinuation (77%)	66.7%
Subbiah V et al. (8)	16	72 (56-85)	ATC	BRAF V600E	Surgery; radiotherapy; chemotherapy; small-molecule targeted therapy	D + T	12-months PFS estimate 79%	Dose reduction (30%); discontinuation (60.5%)	42%
Subbiah V et al. (12)	36	71 (47-85)	ATC	BRAF V600E	Surgery; radiotherapy; RAI; small-molecule targeted therapy; immunotherapy	D + T	6.7	Dose reduction (78%); discontinuation (17%)	56%
Violetis O et al. (19)	1	45	PDTC, Hobnail, and tall-cell PTC	BRAF V600E	Surgery; RAI × 2; lenvatinib + sorafenib; radiotherapy	D + T	24	Discontinuation	Yes
Shimoi T et al. (20)	15	56.5 (16-77)	10 PTC; 5 ATC	BRAF V600E	Surgery; radiotherapy	D + T	5.7	Dose reduction (20%); discontinuation (6%)	45.6%
Jeon Y et al. (21)	27	73 (24-84)	19 PTC; 8 ATC/PDTC	BRAF V600E	Surgery; RAI; lenvatinib + pembrolizumab; sorafenib; chemotherapy	D + T	21.7	Dose reduction (48.1%); discontinuation (81.5%)	44.4%
Tahara M et al. (22)	22	68 (50-77)	17 DTC; 5 ATC	BRAF V600E	Surgery; RAI; radiotherapy; lenvatinib; sorafenib; VEGFR inhibitor	E + B	12-months PFS estimate 78.8%	Dose reduction (13.6%); discontinuation (18.2%)	27.3%

PTC, papillary thyroid carcinoma; ATC, anaplastic thyroid carcinoma; PDTC, poorly differentiated thyroid carcinoma; DTC, differentiated thyroid carcinoma; BRAF V600E, B-Raf proto-oncogene mutation at valine 600; MEK, mitogen-activated protein kinase; ATM, ataxia telangiectasia mutated; TP53, tumor protein p53; TERT, telomerase reverse transcriptase; VHL, Von Hippel-Lindau; MSS, microsatellite stable; TMB, tumor mutational burden; RAI, radioactive iodine; LOF, loss of function; PFS, progression-free survival; VEGFR, vascular endothelial growth factor receptor; D + T, dabrafenib + trametinib; E + B, encorafenib + binimetinib; V, vemurafenib; V + C, vemurafenib + cobimetinib.

## Methods

This case series presents four patients with advanced thyroid cancer: three with PTC and one with ATC, all harboring the BRAF V600E mutation diagnosed using next-generation sequencing (NGS) by The Ion Torrent™ Oncomine™ Dx Express Test in all cases but case 2 in which FoundationOne^®^CDx was used.

Disease progression was assessed using the Response Evaluation Criteria in Solid Tumors (RECIST) ver. 1.1 (15). Treatment-related adverse events were graded using the Common Terminology Criteria for Adverse Events (CTCAE) ver. 5.0 (24). The baseline characteristics, treatment history, efficacy, and safety outcomes were evaluated. The study was approved by the local IRB (TLV-0197-25) and conducted in accordance with the Declaration of Helsinki, with informed consent waived due to its retrospective design.

## Cases presentation

### Case 1

**Figure 2:**
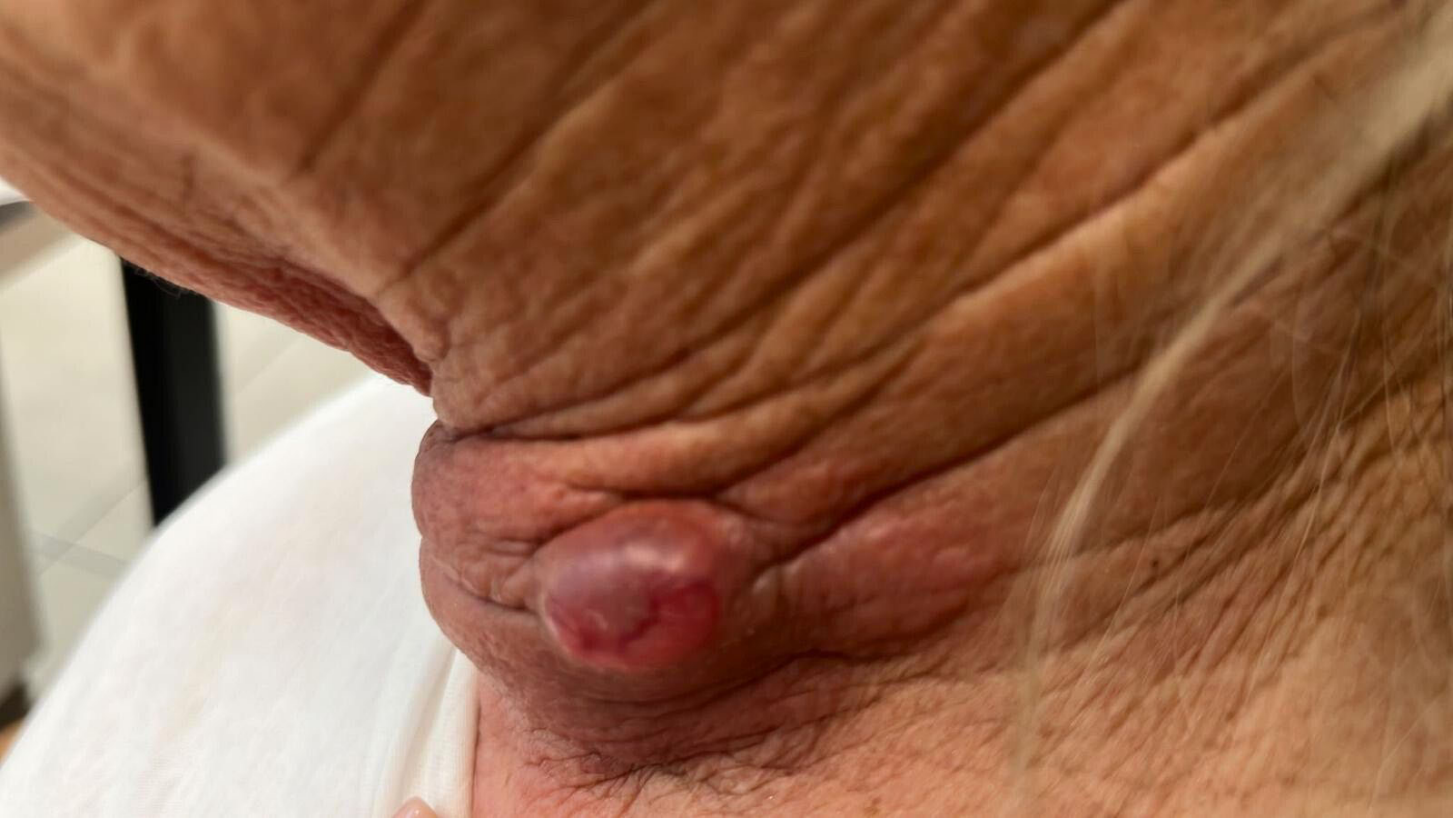
Anaplastic thyroid carcinoma presenting as a neck mass with invasion of the overlying skin. Cutaneous involvement enabled clinical monitoring of the tumor’s response to treatment.

A 68-year-old woman with a history of breast cancer presented in April 2024 with a painful neck mass with cutaneous infiltration (**Figure 2**). Computed tomography (CT) imaging revealed an infiltrative mass extending from the left thyroid lobe to the carotid sheath, displacing the internal jugular vein (IJV) laterally, with a posterior invasion into the larynx and prevertebral fascia. Suspicious cervical lymphadenopathy was also noted. Initial fine needle aspiration (FNA) biopsy was inconclusive between poorly differentiated thyroid cancer and medullary thyroid cancer (MTC). However, subsequent fine-needle biopsy confirmed BRAF-V600E-mutated ATC. A positron emission tomography-computed tomography (PET-CT) demonstrated locally advanced disease without distant metastasis.

She was started on dabrafenib (150 mg twice daily) and trametinib (2 mg once daily), resulting within days in clinical improvement characterized by reduced pain and decreased neck swelling; however, 1 month after treatment initiation, she developed grade 3 encephalopathy that required hospitalization. She presented with fever and an acute confusional state and underwent an extensive diagnostic workup, including lumbar puncture; cerebrospinal fluid analysis, cultures, and PCR tests showed negative results. Discontinuation of therapy led to restoration of her baseline cognitive function. To maintain the therapeutic response that was previously achieved with BRAF and MEK inhibition, she was subsequently started on encorafenib (450 mg once daily) and binimetinib (45 mg once daily) for 4 weeks, but this regimen was discontinued due to disease progression. The patient was then reinitiated on dabrafenib and trametinib, resulting in clinical improvement that persisted for several weeks.

Due to unresectable disease with tracheal infiltration, the patient underwent definitive chemoradiation to the neck, receiving a total dose of 66 Gy in combination with weekly paclitaxel, resulting in a satisfactory clinical and radiographic response. Subsequently, she underwent salvage surgery, which included total thyroidectomy and left neck dissection of levels II–V, with resection of the recurrent laryngeal nerve, IJV, sternocleidomastoid muscle, strap muscles, overlying skin, and shaving of tumor from the laryngeal cartilages and esophageal adventitia. Reconstruction was performed using a pectoralis major flap.

Histopathological examination revealed infiltrating ATC with 50% necrosis, with clean pathological margins. However, 5 weeks postoperatively, the patient developed tracheal necrosis with formation of a tracheocutaneous fistula. Immunohistochemistry of final pathology showed a combined positive score (CPS) of 80 for which she was offered treatment with anti-PD-1 and lenvatinib, which had shown to have some modest benefits as a second-line regimen (25); however, the patient declined further treatment and succumbed to her disease 7 weeks later.

### Case 2

A 67-year-old woman with a history of radiotherapy treatment due to meningioma underwent total thyroidectomy in 2008 for multifocal papillary thyroid cancer (PTC), followed by postoperative RAI therapy (150 mCi). Between 2011 and 2021, she underwent multiple neck dissections for recurrent locoregional disease, detected on sonography, RAI uptake scans, and following PET-CT scans, accompanied by elevated thyroglobulin antibody levels.

In 2021, pathology from a recurrence revealed tall cell variant PTC with mutations in BRAF-V600E and TERT. The disease involved the right recurrent laryngeal nerve (RLN) and was carefully dissected off the internal carotid artery during her most recent surgery. She subsequently received volumetric modulated arc therapy (VMAT) and achieved clinical and radiographic stability for 18 months.

In August 2022, a PET-CT demonstrated new metastases in the lungs and ribs. NGS confirmed positivity for BRAF-V600E, TERT, VHL, microsatellite stability (MSS), and a tumor mutational burden (TMB) of 0. The patient was initiated on lenvatinib (14 mg daily). After 2 months of treatment, a dose reduction to 10 mg daily was necessitated by grade 3 mucositis. Treatment was ultimately discontinued after 14 months of stable disease due to progression, with the development of new skeletal metastases in July 2025.

Treatment with dabrafenib–trametinib was initiated promptly but discontinued early due to severe hypersensitivity with angioedema, rash, and tongue edema requiring corticosteroids and antihistamines. The patient was transitioned to encorafenib–binimetinib with a gradual dose-escalation protocol, starting at 50% of the target dose. On the half-dose regimen, pleural effusion resolved, but escalation was withheld due to weakness and hospitalization for diverticulitis.

**Figure 3:**
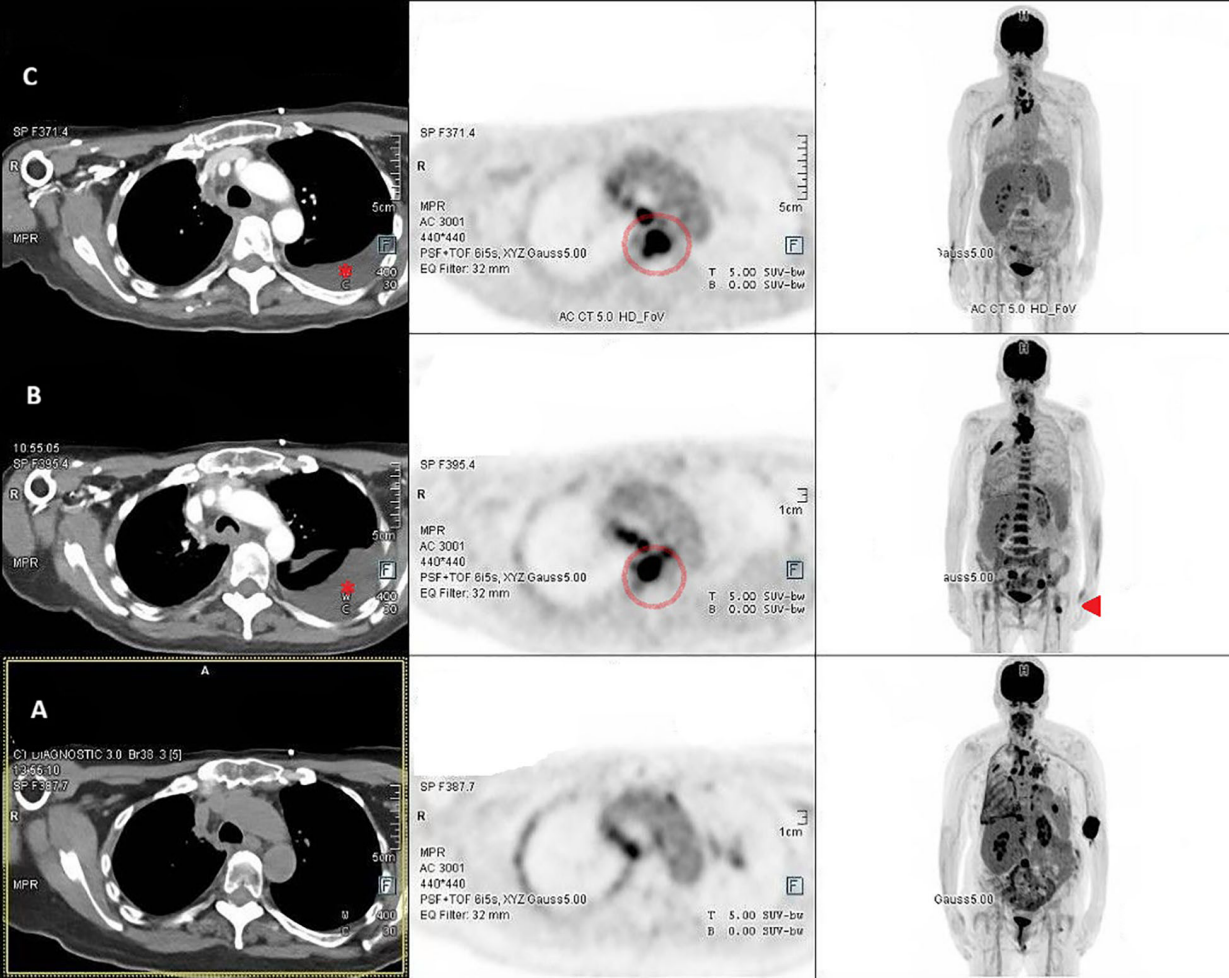
Serial PET-CT images demonstrate the patient’s radiological response and subsequent disease progression. Initial scans [**(A)**, October 2023] show radiological improvement including in the left femur metastasis (arrowhead), following 1 year of therapy on a reduced-dose regimen, coinciding with development and gradual resolution of pleural effusion (asterisk). Later images [**(B)**, April 2024; **(C)**, July 2024] depict disease progression, including the emergence of a D5 vertebral metastasis with associated spinal canal narrowing (circle) and a cervical soft-tissue mass compressing the internal jugular vein. These findings prompted the initiation of cabozantinib therapy. Despite treatment, further progression was observed on follow-up imaging.

Over the course of 1 year, treatment resulted in both clinical and radiological improvement. Subsequently, the patient experienced further local and distant disease progression, including the development of a D5 vertebral metastasis causing spinal canal narrowing and a cervical mass compressing the internal jugular vein (**Figure 3**). This necessitated initiation of cabozantinib at 40 mg daily (reduced dose, adjusted for her frail condition). Despite this intervention, the disease continued to progress, and the patient died 3 months later.

### Case 3

An 83-year-old woman underwent a total thyroidectomy and neck dissection levels II–VI in 2012 for T3N1bM0 PTC. Due to recurrent disease, she received three courses of RAI therapy of 150 mCi each in 2012, 2016, and 2019. In 2022, due radioiodine refractory disease and evidence of mediastinal metastasis, she underwent a selective left neck dissection (level V), with pathology revealing 5 out of 16 positive lymph nodes with extra-capsular extension (ENE). NGS demonstrated a BRAF-V600E mutation.

The patient was initially started on dabrafenib and trametinib but discontinued treatment shortly thereafter due to therapy-induced severe pancreatitis requiring hospitalization. Given evidence of metastatic disease involving the brain, lungs, and liver, treatment was switched to encorafenib (450 mg daily) and binimetinib (45 mg daily), treatment was initiated carefully with gradually dose escalation due to the patient’s age and former acute side effects. However, due to treatment-related severe myalgia that necessitated several brief treatment interruptions, the doses were reduced to encorafenib 150 mg once daily and binimetinib 15 mg once daily.

Despite dose modifications, thyroglobulin levels remained stable at approximately 1,100 ng/mL and brain metastasis remained stable on MRI imaging. After 8 months, the patient elected to discontinue active therapy due to persistent myalgia. In January 2025, dabrafenib–trametinib was reintroduced at half the standard dose under close monitoring of amylase and lipase levels, without recurrence of pancreatitis. The patient continues to demonstrate clinical improvement, with thyroglobulin levels decreasing from 7,516 to 1,764 ng/mL as of July 2025.

### Case 4

A 76-year-old man presented in November 2020 with a thyroid mass infiltrating the trachea, as well as regional and distant metastases to the lungs, mediastinum, scapula, third rib, and L1 vertebra. The patient underwent a total thyroidectomy with a right lateral neck dissection in February 2021. Tracheal resection was performed for tumor extension, leaving positive surgical margins to avoid the need for total laryngectomy. Pathology revealed tall cell variant PTC, and 8 out of 40 lymph nodes were positive for disease with minimal ECE.

Postoperatively, he received 150 mCi RAI therapy in March 2021. NGS demonstrated mutations in BRAF-V600E and TERT. Dabrafenib–trametinib was initiated in April 2021, but therapy was soon discontinued due to acute liver injury. He was transitioned to encorafenib (450 mg QD) and binimetinib (45 mg QD), achieving a partial response with a thyroglobulin decrease from 20,000 to 8,000 and radiographic and metabolic disease regression on PET-CT. However, treatment was discontinued after 4 months due to acute kidney injury associated with capillary leak syndrome.

To maintain systemic therapy, the patient was initiated on a third BRAF and MEK inhibitor combination, vemurafenib (360 mg BID) and cobimetinib (40 mg QD), approximately 16 months after diagnosis. The disease remained stable for 15 months but showed oligometastatic progression at 21 months, with new skeletal metastases involving the vertebrae and hips. Vemurafenib was escalated to the standard dose; however, follow-up PET-CT imaging 6 months later confirmed further disease progression. He received radiation to progression sites and lenvatinib was introduced at 25 months, but the disease continued to advance, culminating in the patient’s death at 31 months.

## Discussion

Here, we presented four cases of thyroid cancer, three PTC and one ATC, which highlight the complex nature of managing advanced thyroid cancers, particularly those with BRAF V600E mutations. All patients received standard-of-care treatments, including surgery, radiation, radioactive iodine, and multikinase inhibitors. For progressive locoregional disease, various BRAF–MEK inhibitors were used as personalized salvage therapy to delay progression and improve quality of life, obtained through individual insurance approvals since they are not included in standard protocols.

**Figure 4:**
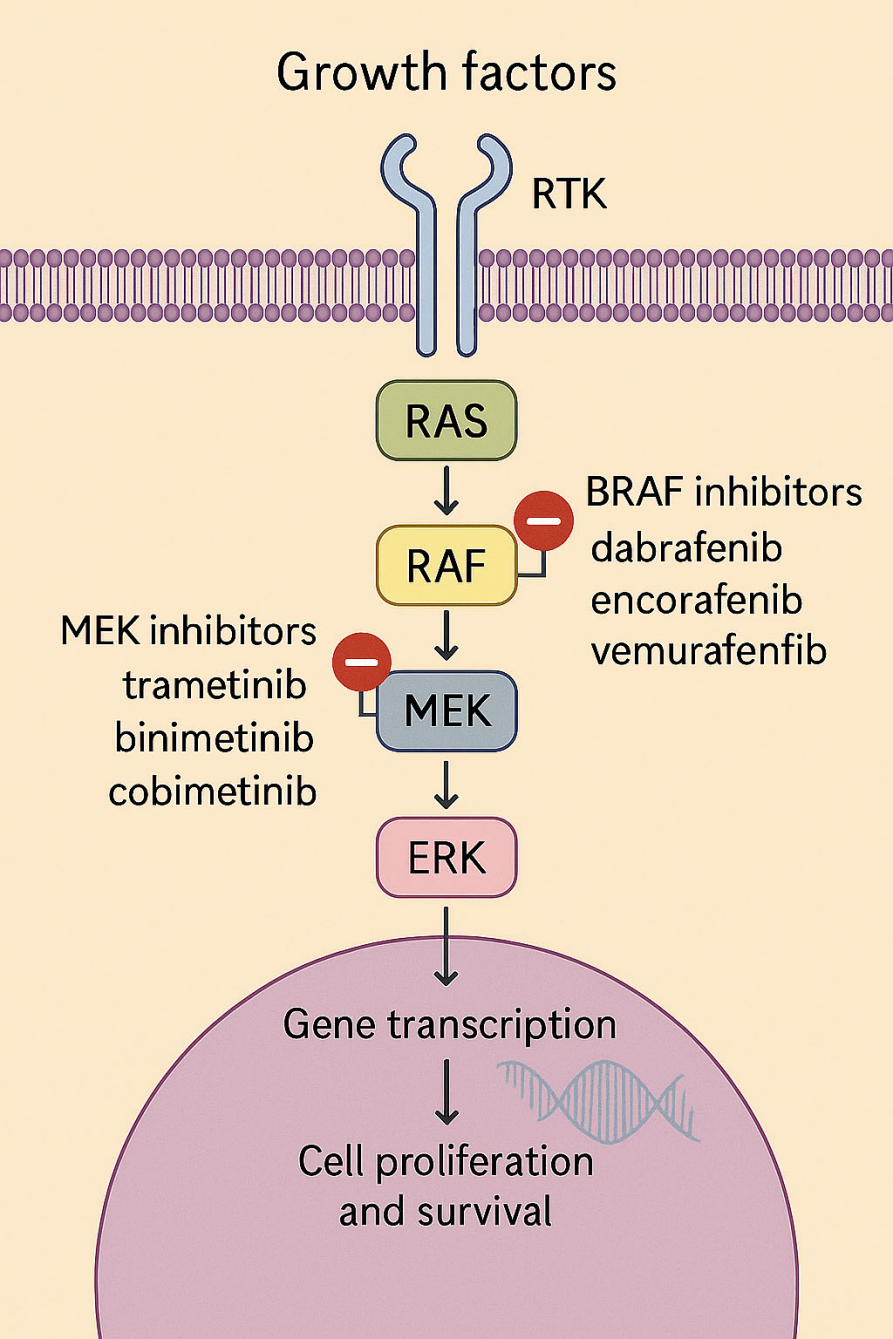
Schematic representation of the MAPK/ERK signaling pathway and sites of targeted inhibition. Binding of growth factors (GF) to receptor tyrosine kinases (RTKs) at the cell membrane activates the RAS–RAF–MEK–ERK cascade, ultimately promoting gene transcription, cell growth, and survival. Mutant BRAF (V600E/K) leads to constitutive activation of the pathway. Targeted therapies include BRAF inhibitors: dabrafenib, encorafenib, and vemurafenib, and MEK inhibitors: trametinib, cobimetinib, and binimetinib, which act at their respective kinase levels to block downstream signaling.

The BRAF V600E mutation constitutively activates the mitogen-activated protein kinase (MAPK) pathway by promoting ligand-independent phosphorylation of downstream kinases MEK and ERK (9, 13). This dysregulation promotes uncontrolled cell growth and differentiation, contributing to oncogenesis of tumor cells (16, 17). Targeted inhibition of BRAF and MEK interrupts this signaling cascade and has demonstrated clinical benefit across several BRAFV600E-mutated solid tumors (**Figure 4**) (18).

The identification of BRAF V600E mutations in these cases underscores the critical role of molecular profiling in guiding treatment decisions (23), particularly in heavily pretreated patients. BRAF V600E is a known driver of thyroid cancer progression and a target for therapy (5, 26, 27). BRAF and MEK inhibitor combinations such as dabrafenib–trametinib, encorafenib–binimetinib, and vemurafenib–cobimetinib exemplify the transition toward personalized medicine in advanced thyroid cancer (14, 23).

While multiple BRAF–MEK combinations are approved for melanoma, with comparable efficacy and slightly differing toxicity profiles, approval in thyroid cancer remains more limited (28). As of 2025, dabrafenib plus trametinib is the only FDA-approved targeted regimen for unresectable or metastatic BRAF V600E-mutant ATC, based on the ROAR trial, which demonstrated a 61% response rate (10–12). This combination later received tumor-agnostic approval for BRAF V600E-mutant solid tumors and is recommended by ESMO as standard therapy for eligible ATC patients (10, 12, 18, 29).

Earlier ATA guidelines (2015) recommended kinase inhibitors for progressive, symptomatic, radioiodine-refractory differentiated thyroid cancer; however, BRAF–MEK combinations were not included due to limited evidence (30–32). The updated 2025 ATA guidelines now support BRAF-directed therapy as a first-line option for patients unsuitable for lenvatinib or those who progress or develop intolerance to prior kinase inhibitors (33).

Toxicity remains a major limitation of BRAF–MEK inhibition. All four patients in this series (100%) developed grade ≥3 adverse events requiring dose modification or discontinuation. The most common toxicities were pyrexia (100%), myalgia (75%), gastrointestinal intolerance (75%), and hepatotoxicity (50%). Given the high incidence and severity of adverse events, rechallenge with BRAF–MEK inhibitors should be cautiously considered, particularly in frail or elderly patients. In case 1, reintroduction of dabrafenib–trametinib following prior encephalopathy and disease progression on encorafenib–binimetinib led to a transient partial response.

Similar toxicity rates are reported in clinical trials. In the phase II ROAR study, 100% of patients experienced adverse events on the standard dabrafenib (150 mg BID) and trametinib (2 mg daily) regimen, with 58% having grade 3–4 toxicity and half requiring dose interruption or reduction (12). Similar toxicity patterns were reported in melanoma, as shown by Long et al., where treatment with dabrafenib and trametinib for stage III BRAF-mutated melanoma resulted in adverse events in 97% of patients, including 41% with grade 3–4 events that led to dose interruption, dose reduction, and treatment discontinuation in 66%, 38%, and 26% of cases, respectively (34). Although large phase III randomized controlled trials evaluating BRAF and MEK inhibitors in thyroid cancer are lacking, case reports, systematic reviews, and meta-analyses of early-phase and non-randomized studies have demonstrated meaningful clinical activity, manageable safety profiles, and improved outcomes in this historically aggressive and treatment-refractory population (12, 14, 15, 17–19, 35, 36). In other settings, trials in advanced RAI-resistant differentiated thyroid cancer have shown the feasibility of using BRAF and MEK inhibitors as part of a redifferentiation treatment strategy, thereby broadening the therapeutic potential of these drug combinations (25, 37).

In a phase II trial by Marcia S. Brose et al. (7), the efficacy of vemurafenib was evaluated in 51 patients with metastatic or unresectable BRAF V600E–positive, RAI-refractory PTC. Vemurafenib achieved a partial response in 38% of patients who had not received prior VEGFR inhibitor therapy, compared with 27% among those previously treated with a VEGFR inhibitor. The corresponding median PFS was 18.2 months versus 8.9 months, respectively. The lower response rates observed in both cohorts may be attributed to the use of BRAF inhibitor monotherapy without concurrent MEK inhibition, and to the latter cohort being more heavily pretreated and having received multiple prior systemic therapies, including chemotherapy, which is rarely used in current practice. Grade 3–4 adverse events occurred in approximately two-thirds of patients, with no treatment-related deaths reported.

Extrapolating from melanoma experience, alternative BRAF and MEK inhibitor combinations beyond dabrafenib–trametinib may help mitigate toxicity while maintaining efficacy in advanced thyroid cancer. In our series, following severe adverse events with two prior regimens, switching to vemurafenib–cobimetinib in case 4 achieved 14 months of stable disease, underscoring the potential benefit of strategic sequencing of alternative targeted combinations.

**Figure 5:**
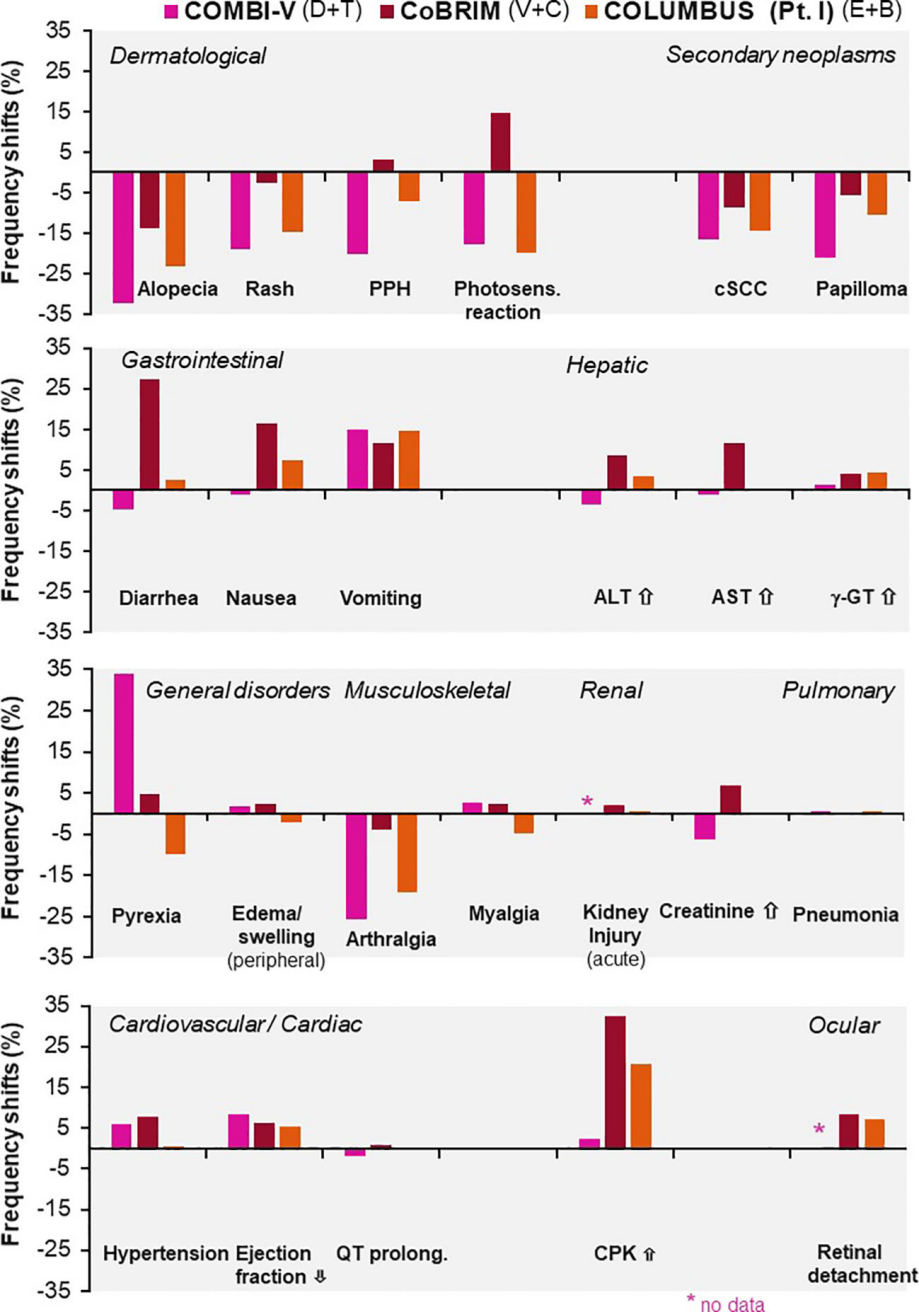
Differences in adverse event frequencies between combination therapies tested for advanced melanoma in three major clinical trials: COMBI-V (dabrafenib plus trametinib), CoBRIM (vemurafenib plus cobimetinib), and COLUMBUS Part 1 (encorafenib plus binimetinib). Shown are frequency shifts (%) in selected adverse events observed in patients treated with. Positive values indicate increased frequency relative to monotherapy; negative values indicate decreased frequency. Reprinted from ESMO Open, Vol. 4, Davies MA et al., “Comparison of adverse event profiles of MEK inhibitors across indications: a review of clinical trials,” e000491, Copyright (2019), with permission from Elsevier.Sciwheel inserting bibliography.

Although these combinations share overlapping toxicities, each agent has a distinctive adverse effect profile: vemurafenib–cobimetinib is associated with photosensitivity and ocular toxicity; dabrafenib–trametinib with pyrexia; while encorafenib–binimetinib with relatively lower pyrexia risk. These differences in adverse effects were thoroughly evaluated in three major clinical trials for advanced melanoma and previously published in *ESMO Open*, as illustrated in **Figure 5** (38). Notably, encephalopathy is a rarely reported adverse effect; however, in case 1, it developed during treatment and, following thorough neurological evaluation, was attributed to dabrafenib and trametinib therapy.

Despite initial responses to targeted therapies, all cases eventually showed disease progression, indicating the development of treatment resistance. Resistance due to escape phenomenon is well-documented in BRAF-mutated cancers, develops faster in thyroid cancer relatively to BRAF positive melanomas, and underscores the need for sequential treatment strategies and ongoing research into resistance mechanisms (24, 32). Reported mechanisms include reactivation of the MAPK pathway through NRAS, KRAS, or CRAF mutations, enhanced receptor tyrosine kinase signaling (HER2, HER3, MET), and secondary alterations in MEK or BRAF splice variants (39, 40). Tumor heterogeneity and clonal evolution further limit response durability (41). These findings emphasize the need for re-biopsy and molecular re-profiling at progression to inform second-line strategies or trial enrollment.

Two patients (50%) in our series harbored concomitant TERT promoter mutations. Although BRAF and TERT co-mutations are well recognized for their association with radioiodine resistance and more aggressive disease (42), the limited sample size in our cohort precludes definitive conclusions regarding their role in treatment resistance. Further studies are warranted to elucidate their potential contribution to resistance to BRAF and MEK inhibitors.

In the updated phase II ROAR trial, the objective response rate (ORR) was 56% with a median progression-free survival (PFS) of 6.7 months (12). The Japanese BELIEVE trial demonstrated a lower ORR of 33.3% and median PFS of 5.7 months in a cohort comprising 10 PTC patients and 5 with undifferentiated thyroid cancer, without distinction between poorly differentiated carcinoma (PDC) and ATC (20). In our series, progression-free survival for dabrafenib trametinib averaged 5.75 months (based on two evaluated patients), 5.75 for encorafenib–binimetinib (based on four evaluated patients), and 14 for vemurafenib–cobimetinib (based on one evaluated patient).

Superior outcomes were reported by the Korean group of Jeon et al. (2024), with a total ORR of 73.1% and differential PFS outcomes of 23.3 months for PTC and 4.5 months for undifferentiated histologies (21). Notably, this improved efficacy may be attributed to the study design, wherein out of the 27 patients two-thirds of patients received dabrafenib plus trametinib as first-line therapy, without prior exposure to cytotoxic chemotherapy or multiple kinase inhibitors. An additional small phase 2 trial including 22 patients, of whom 17 had differentiated thyroid cancer and 9 ATC, found that encorafenib and binimetinib achieved an ORR of 47.1% in the differentiated subgroup and 54.5% overall (95% CI 32.2–75.6), with partial responses in 12 patients and stable disease in 10 (22). Grade 3 adverse events occurred in 27.3%, with no grade 4 or 5 events, highlighting a modest safety profile (22).

Case 1 illustrates the therapeutic potential of BRAF-directed therapy in ATC, historically associated with dismal prognosis (4, 43). The use of BRAF-targeted therapy in this case aligns with recent advancements in ATC treatment, offering a potential avenue for improved outcomes in this historically difficult-to-treat malignancy (26, 27, 29, 44). Treatment with BRAF+MEK inhibitors as well as the initiation of chemoradiation therapy not only saved the patient from undergoing total laryngectomy but also made the tumor operable with the opportunity of achieving clear surgical margins. Unfortunately, the tumor recurred and the patient refused further treatment even though the last pathology confirmed a combined positivity score of 80.

In case 3, BRAF–MEK inhibition demonstrated intracranial activity in brain metastases, consistent with literature reporting adequate central nervous system penetration over multiple kinase inhibitores (45). Although lenvatinib is considered as the first-line treatment for RAI-refractory PTC (33), the use of BRAF and MEK inhibitors is often preferred in the immediate postoperative period following major surgery because of the risk of impaired wound healing (46). This consideration was the primary rationale for selecting BRAF and MEK inhibitor regimen in case 4, after the patient had undergone tracheal resection.

## Conclusion

These cases underscore the necessity of a multimodal, individualized approach to advanced thyroid cancer. The integration of surgery, radiation, radioiodine, and targeted systemic therapy optimizes disease control, while molecular profiling has become indispensable in guiding precision treatment. BRAF and MEK inhibition represents a major therapeutic advancement; however, toxicity management, treatment sequencing, and the emergence of resistance remain significant challenges. This case series highlights both the progress achieved and the unmet needs in managing advanced thyroid malignancies. Continued clinical investigation and translational research are essential to refine patient selection, enhance tolerability, and improve long-term outcomes in this complex disease spectrum.

## Data Availability

The original contributions presented in the study are included in the article/supplementary material. Further inquiries can be directed to the corresponding author.
